# **Pharmacological** Aspects of *Vipera* *xantina* *palestinae* Venom

**DOI:** 10.3390/toxins3111420

**Published:** 2011-11-14

**Authors:** Tatjana Momic, Franziska T. Arlinghaus, Hadar Arien-Zakay, Jeoshua Katzhendler, Johannes A. Eble, Cezary Marcinkiewicz, Philip Lazarovici

**Affiliations:** 1 School of Pharmacy Institute for Drug Research, Faculty of Medicine, The Hebrew University of Jerusalem, Jerusalem 91120, Israel; Email: momict@gmail.com (T.M.); hadara@ekmd.huji.ac.il (H.A.-Z.); katzhe@cc.huji.ac.il (J.K.); 2 Center for Molecular Medicine, Department of Vascular Matrix Biology, Frankfurt University Hospital, Excellence Cluster Cardio-Pulmonary System, 60590 Frankfurt, Germany; Email: Arlinghaus@med.uni-frankfurt.de (F.T.A); eble@med.uni-frankfurt.de (J.A.E.); 3 Department of Biology, Temple University College of Science and Technology, Philadelphia, PA 19122, USA; Email: cmarcink@temple.edu

**Keywords:** *Viperaxantinapalestinae*, venom, neurotoxin, hemorrhagin, integrin inhibitors, antivenom

## Abstract

In Israel, *Vipera xantina palestinae* (*V.x.p.*) is the most common venomous snake, accounting for several hundred cases of envenomation in humans and domestic animals every year, with a mortality rate of 0.5 to 2%. In this review we will briefly address the research developments relevant to our present understanding of the structure and function of *V.x.p.* venom with emphasis on venom disintegrins. Venom proteomics indicated the presence of four families of pharmacologically active compounds: (i) neurotoxins; (ii) hemorrhagins; (iii) angioneurin growth factors; and (iv) different types of integrin inhibitors. Viperistatin, a α1β1selective KTS disintegrin and VP12, a α2β1 selective C-type lectin were discovered. These snake venom proteins represent promising tools for research and development of novel collagen receptor selective drugs. These discoveries are also relevant for future improvement of antivenom therapy towards *V.x.p.* envenomation.

## 1. Introduction

Snake bite is a serious medical problem, particularly in Asia, Africa and in the Middle East, including Israel. In Israel, *Vipera xantina palestinae* (*V.x.p.*) is the most common venomous snake accounting for 100-300 reported cases of envenomation in adults, children and domestic animals every year [1] with a mortality rate of 0.5 to 2% [2,3]. The main symptoms following envenomation include hemorrhagic activity manifested in edema, alteration in the coagulation system, myotoxicity resulting in massive muscle fiber collagen desheeting and myonecrosis, cardiotoxicity and neurotoxicity often leading to strong hypotension, arrythmic pathologies, flaccid paralysis, disregulation of central autonomic vasoregulatory mechanism and respiratory paralysis [4]. The *V.x.p.* venom contains enzymes such as hyaluronidases, esterases, phosphodiesterases and L-amino acid oxidases [5] as well as neurotoxic phospholipase A_2_, and hemorrhagins. The above clinical local and systemic symptoms of *V.x.p.* envenomation are the consequence of the pharmacological activity of these enzymatic and non-enzymatic venom proteins. They cause increased capillary permeability, endothelial damage, platelet aggregation and dysfunction, thromboplastin and thrombin inhibition, neutrophilia, leucocytosis, thrombocytopenia, increase fibrinolysis and hypofibrinogenemia, release of histamines, kinins, and different presynaptic neurotoxic effects [6,7]. These pathological syndromes are induced by the large variety of proteins found in *V.x.p.* venom and by additive and synergistic interactions between them.

In this review we will briefly address the research developments relevant to our present understanding on the structure and function of venom components of *V.x.p.* with emphasis on integrin inhibitors. These considerations are also relevant for future improvement of antivenom therapy towards *V.x.p.* envenomation.

## 2. *V.x.p.* Venom Active Components

### 2.1. Neurotoxins

Isolation of neurotoxic and hemorragic factors from *V.x.p.* venom started in the 50s and 60s using chromatographic methods available at that time. Several toxic fractions were isolated and characterized from the venom of *V.x.p.* [8]. One of them was further isolated by Moroz-Perlmutter *et al*., who demonstrated its lethality to mice and synergistically induced neurotoxicity with the venom protease fraction [9]. Later, Ovadia *et al*. [10] found that the neurotoxic fraction is composed of two proteins: an acidic protein (pI 4) endowed with phospholipase A_2_ (PLA_2_) (EC3.1.1.4) activity and a basic protein (pI 9.5) lacking any known enzymatic activity. Each of these proteins when injected into mice were not lethal but when injected together intravenously induced neurotoxicity with LD_50_ between 50 and 100 µg/kg in mice [10]. These studies were continued by Bdolah and coworkers who demostrated that the acidic phospholipase is toxic and that substitution of this phospholipase with other phospholipases from different snakes can cause lethality in mice upon intavenous coinjection with the non-enzymatic basic component [11]. In the mid-90s, Krizaj *et al.* cloned the acidic PLA_2_ from *V.x.p.* (*Vpa*PLA_2_) which has an apparent MW of 15 kDa, consisting of 122 amino acids and belonging to the subgroup IIA of PLA_2s_[12]. The neurotoxic mechanism of the two component system of the *V.x.p.* venom is not clear and the structure of the basic protein is yet unknown. Future studies are required to characterize the interaction between *Vpa*PLA_2_ and its basic counterpart including their unusual synergistic toxicity.

### 2.2. Hemorrhagins

In the early 60s, the group of De Vrise and Moroz isolated a hemorrhagic fraction from *V.x.p*. which is an acidic protein with an estimated MW of 44 kDa [13,14]. Later, Ovadia isolated three hemorrhagic factors from the *V.x.p.* venom, two of them with strong proteolytic activity on gelatin and casein as well as a capillary permeability-increasing albeit non-proteolytic activity, all of them in the range of 60 kDa MW [15]. In continuation to these studies Nakar and associates separated a proteolytic enzyme from one of the hemorrhagins. The two other hemorrhagins were endowed with proteolytic activity which could not be chromatographically separated from the hemorrhagic activity [16]. This strongly supported the concept that certain capillary permeability factor(s), devoid of proteolytic activity as well as several metalloproteases represent the hemorrhagins originally identified by Grotto *et al*. [14]. There has been a 25-year gap between these former chemical and pharmacological studies and nowadays, when more modern and advanced proteomic and pharmacological characterizations have approached to re-evaluate *V.x.p.* venom. 

### 2.3. Proteomics

**Figure 1:**
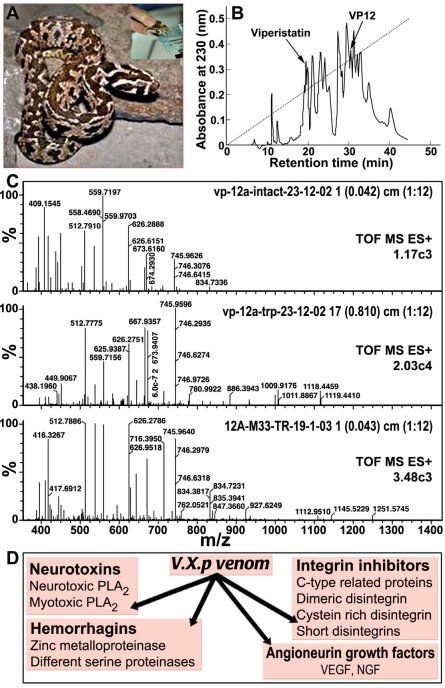
Scheme of the steps followed in the venomic investigation of *Vipera xantina palestinae* (*V.x.p*.). (**A**) Photograph of *V.x.p.* snake and manual milking of snake venom (Insert); (**B**) representative separation of venom components viperistatin and VP12 on C_18_ reverse phase high-performance liquid chromatography (HPLC); (**C**) typical mass spectroscopy fragmentation spectra of isolated HPLC VP12A; (**D**) Scheme of the major groups of pharmacologically active protein in *V.x.p.* venom.

A preliminary proteomic analysis of *V.x.p.* venom is presented in Figure 1. The *V.x.p.* snakes, kept in a serpentarium in compliance with animal welfare regulation, were gently milked under good laboratory practice conditions (Figure 1A). The liquid venom was lyophilised and 200 mg dried venom was separated by C_18_ reverse phase HPLC into 17 fractions (Figure 1B). The fractions showing a single electrophoretic band (with or without additional separation by HPLC), were submitted for molecular mass, and *N*-terminal sequence determination, and mass spectrometry analysis as presented for VP12A (Figure 1C). The partial sequences of the proteins were assigned by BLAST analysis to known families of snake venom proteins. Although only few toxins from any particular reptile species are annotated in the databases, representative members of most toxin families of other snake species are available in the UniProtKB/Swiss-Prot v56.5 database [17] allowing the identification of the searched *V.x.p.* sequences. The analysis of *V.x.p.* venom HPLC fractions performed by MALDI-TOF indicated the presence of complex mixture of pharmacologically active molecules representing different percentage of whole venom according to the following distribution: (i) **neurotoxins**: 2% neurotoxic PLA_2_; 2% myotoxic PLA_2_; (ii) **hemorrhagins**: 65% zinc metalloproteinase, 9% different serine proteinases; (iii) **angioneurin growth factors**: about 2% of the venom is composed of snake homologues of vascular endothelial growth factor (VEGF) [18] and nerve growth factor (NGF) known to induce angiogenesis in blood capillaries, neurite outgrowth, as well as vascular permeability [19,20] and functionally also assigned to the hemorrhagin family; (iv) **integrin inhibitors**: 10% C-type lectin-related proteins (CLRPs), 6% dimeric disintegrin, 1% cystein rich disintegrin, <1% short disintegrins (hypothesized to represent additional hemorrhagins) [21]; (Figure 1D). This *V.x.p.* venom proteomics is in-line with snake venomics of other Vipera venoms, indicating a very similar composition [22]. It is evident that Vipera snakes produce a complex mixture of a large number of distinct proteins that pathologically modulate the cardiovascular and nervous system. In spite of the fact that viperid venoms may contain over 100 protein compounds, these proteins can be sorted into **enzymes** (serine proteinases, zinc-metalloproteases, L-amino acid oxidase, group II PLA_2_) and **proteins without enzymatic activity**, such as disintegrins, C-type lectin-related proteins (CLRPs), natriuretic peptides, myotoxins, cysteine-rich secretory protein (CRISP) toxins, nerve and vascular endothelium growth factors, cystatin, and Kunitz-type protease inhibitors [22]. This situation may reflect the fact that these proteins evolved from a restricted set of gene protein families with normal, physiological functions that were modulated to serve a variety of novel pathologically offensive functions such as to induce neurotoxicity, hemorrhages, and muscle damage, thereby immobilizing and digesting the prey. This proteomic information requires further proof by biochemical and pharmacological studies of all HPLC isolated proteins both *in vitro* and in animal models.

## 3. *V.x.p.* Growth Factors: Vascular Endothelial Growth Factors (VEGF) and Nerve Growth Factor (NGF)

Vascular endothelial growth factors (VEGF) are a family of proteins divided into seven subtypes: mammalian VEGF-A, B, C, D, placenta growth factor (PlGF), viral VEGF (VEGF-E), and snake venom VEGF (VEGF-F) [18]. The most characteristic structural feature of these VEGF ligands is the cysteine knot motif comprising three intertwined disulfide bridges. To date, only five receptors have been identified for the growing number of ligands [23]. Three belong to the receptor tyrosine kinase (RTK) family and are called *fms*-like tyrosine kinase-1 (Flt-1, also known as VEGFR-1) [24,25], kinase insert domain- containing receptor (KDR, VEGFR-2) [26,27], and Flt-4 (VEGFR-3) [28,29,30]. The others are non-tyrosine kinase-type receptors neuropilin-1 (NP-1) and neuropilin-2 (NP-2), which are believed to function as co-receptors for some VEGF subtypes and their isoforms [31,32]. Over ten different VEGF-F proteins have been identified. Snake venom VEGFs also show different degrees of selectivity in their interactions with different receptors and therefore have been divided into three groups [18]. VEGF-F_1_ is very selective for VEGFR-2, VEGF-F_2_ predominantly binds to VEGFR-1 and weakly to VEGFR-2, and VEGF-F_3_ binds to both VEGFR-1 and VEGFR-2, as well as to NP-1. Snake venom VEGFs display potent endothelial cell proliferation, hypotensive activities, and vascular permeability induction when compared with VEGF-A_165_, the most physiologically abundant and investigated isoform of the VEGF-A family [33].

We have recently isolated and characterized VEGF-like angioneurin compounds from *V.x.p.* venom using two steps of reverse phase HPLC followed by protein sequencing yielding the following primary structure:

ZVRPFLDVYQRSACQARETLVSILQEYPDEISDIFRPSCVAVLRCSGCCTDESLKC

TPVGKHTVDMQIMRVNPRTQSSKMEVMKFTEHTACECRPRRKQGEPDGPKEKPR

As expected, this growth factor [18] was able to induce *in vivo* plasma extravasation by causing microvessel leakage and *in vitro* increased permeability (reduction in electrical resistance) of brain capillary endothelial monolayer (Lecht, S.; Cohen, G.; Marcinkiewicz, C.; Lazarovici, P. 2008, unpublished data). These properties strongly suggest that this growth factor is another member of the hemorrhagin group of proteins in *V.x.p.* venom. The vipera VEGFs, like the natural human ligand VEGF-A_165_ exert their specific activity by binding to a VEGFR-2 but do not bind to the Flt or neuropilin receptor families [33]. For the first time unique pharmacological tools are available to study VEGF receptor type 2 function and for development of novel agonists and antagonists.

Nerve growth factor (NGF) is a polypeptide which belongs to the neurotrophin family. The neurotrophin family includes four members: NGF, brain-derived neurotrophic factor (BDNF), neurotrophin3 (NT-3) and neurotrphin4/5 (NT-4/5) [34]. NGF plays the crucial role in the sympathetic and sensory nervous systems [35]. The biological functions of NGFs are mediated by two classes of cell surface receptors: the p75 neurotrophin receptor (p75^NTR^), which recognizes all members of the neurotrophin family, and the tropomyosine kinase related receptor (trkA), belonging to the tyrosine kinase-neurotrophin receptor family [36]. Many NGFs have been isolated and characterized from all three families of snakes: Elapidae, Viperidae, Crotalidae [37,38,39]. We have initiated isolation and purification of NGF from *V.x.p.* venom. This factor also induced weak vascular permeability suggesting its involvement in the hemorrhagic activity of *V.x.p.* venom (data not shown).

Similar to snake venom VEGF-induced angiogenic effects, venom-derived NGFs promoted migration [40], capillary sprouting and other angiogenic functions [41] and may serve as unique pharmacological tools to study NGF actions.

## 4. *V.x.p.* Integrin Inhibitors

Disintegrins are the best known naturally occurring antagonists of integrins. Integrins are cell surface receptors, heterodimers of α and β subunits. Their main function is to shape and maintain the proper structure of tissue by mediating cell-cell and cell-extracellular matrix interactions. Besides their structural function, integrins have been identified as signaling molecules [42], resulting in cytoskeleton reorganization (shape change, adhesion, migration) [43,44,45], regulation of cell proliferation and cell survival and apoptosis [45,46,47].

Currently, classification of snake venom disintegrins can be performed based on their structure and function. The structural classification proposed by Marcinkiewicz presents two groups of disintegrins: monomeric and dimeric. Monomeric disintegrins can be divided into three subclasses regarding the number of cysteins in their structure: short, medium and long [48].

Functionally, disintegrins can be divided into three groups according to their integrin selectivity and presence of specific, recognition motifs. This classification includes RGD-disintegrins (binding to αIIbβ3, αvβ3, α5β1 integrins which recognize fibrinogen, vitronectin and fibronectin), MLD-disintegrins (binding to α4β1, α4β7 integrins which binds to fibronectin, VCAM-1, MadCAM-1, and binding to α9β1 integrin which preferentially interacts with tenascin-C, osteopontin, VCAM-1, fibronectin, trombospondin-1, ADAM family members, and growth factors such as VEGF and NGF), and KTS-disintegrins (binding to the α1β1 integrin, a collagen receptor with particularly good affinity for collagen IV) [48,49].

**Figure 2:**
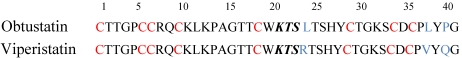
Comparison of the amino acid sequences of disintegrins: viperistatin (*V.x.p.* [21]) and obtustatin (*V. lebetina obtusa* [51]). Cysteines are in red and the active motifs are in italic. Amino acid residues that are different in obtustatin and viperistatin are in blue.

Viperistatin, a KTS-disintegrin protein was isolated from *V.x.p.* venom by Kisiel *et al*. [21] using two-step reverse-phase high-performance liquid chromatography (HPLC) [50]. Viperistatin is a peptide of 4454.5 Da, containing eight cysteines residues engaged in the formation of four disulfide bonds. The complete primary structure of viperistatin determined by *N*-terminal sequencing showed high homology of this peptide to obtustatin, another KTS-containing disintegrin (Figure 2) [51], differing from it in just three residues at positons 24, 38 and 40. Viperistatin contains the same KTS motif in its active site as obtustatin and shows α1β1 integrin-blocking activity as well. However, it appeared to be a much more potent inhibitor of α1β1 integrin then obtustatin. In adhesion assay, viperistatin was 25 fold more active in inhibition of integrin α1 transfected K562 cell adhesion to both collagen IV and collagen I. The same trend was observed when inhibitory activity of viperistatin and obtustatin was compared in ELISA, where binding of purified α1β1 integrin to immobilized collagen type IV was investigated [21]. Structure-function analysis using synthetic, linear peptides that corresponded to the amino acid sequence of the entire integrin-binding loop for both obtustatin (CW**KTS**LTSHYC) and viperistatin (CW**KTS**RTSHYC), in adhesion and ELISA assay, indicated that replacement of a leucine (obtustatin) for an arginine (viperistatin) increased the inhibitory activity towards α1β1 integrin by 6 to 10 fold [21] or even 25 fold [52] (Figure 2). The partial relative enhancement of the inhibitory activity of the KTSR-peptide suggested that the L^38^/V and P^40^/Q substitution of viperistatin may also contribute to the biological activity of this disintegrin [21,52,53].

A C-type lectin-related protein (CLRP), called VP12 was also isolated from *V.x.p.* venom by Staniszewska *et al*., using two-step reverse-phase HPLC [54]. The amino acid sequence of VP12 subunits were established by a combination of mass spectrometry and *N*-terminal sequencing of their proteolytic fragments. While VP12A subunit sequence is completed, the VP12B subunit sequence is partial and needs to be completed and confirmed by DNA sequencing. MALDI-TOF MS analysis of unmodified VP12 yielded a single molecular ion of 30.387 Da, whereas VP12A and VP12B subunits showed 15.981 Da and 15.893 Da, respectively. Based on calculations and by comparison with the structure of another C-lectin type protein EMS-16 and rhodocetin [55,56] which have similar activity to VP12, it was predicted that each subunit of VP12 contains seven cysteines [54]. Although a large number of CLRP family proteins were reported, only few of them exhibit integrin binding properties [57]. In adhesion assay, VP12 showed a potent inhibitory effect on the adhesion of integrin α2β1 overexpressing K562 cells to collagen I (IC_50_ 0.5 nM), but not on K562 cells transfected with α1 integrin subunit to collagen IV. Also, the direct interaction of VP12 with α2β1 integrin was confirmed in adhesion assay. Cells transfected with the α2 integrin subunit showed a potent, dose-dependent adhesion to immobilized VP12, whereas non-transfected control cells did not interact with this CLRP [54]. This feature of VP12 was used for establishing of a technique for VP12 isolation from *V.x.p.* venom, based on affinity chromatography targeting the A-domain of the α2β1 integrin [58].

By virtue of selective inhibiton of α1β1 or α2β1 integrins, respectively, viperistatin and VP12 are also active in blocking experimental metastasis of melanoma cell clones expressing these individual collagen receptors. This was shown in *in vitro* and *in vivo* experiments of inhibitory effects of both disintegrins on: adhesion of melanoma cells to collagen I and IV [58], transmigration of human melanoma cells through primary endothelial dermal human microvascular endothelial cells (dHMVEC) monolayers, as well as melanoma metastasis in a B16F10 mouse model [54,59]. Viperistatin and VP12 demonstrated antimetastatic activity after injection into the tail vein of the mice at a dose lower than 5 mg/kg, a concentration which is not toxic to the animals.

All these studies provide KTS-peptides as lead compounds for the development of selective α1β1 integrin antagonist drugs. Establishment of the full sequence of VP12 and elucidation of the binding motif of VP12 to α2β1 integrin would also offer the possibility to develop and synthesize cyclic peptides with α2β1 integrin-inhibiting potential. Such collagen receptor inhibitors would enable the design of a variety of drugs towards therapy of different cardiovascular diseases and cancer.

## 5. Antivenom Therapy of *V.x.p.* Envenomation

Serotherapy is currently the main therapy for treating snake envenomation in general and *V.x.p.* in particular. In Israel, *V.x.p.* bites are among the most common reasons for human and veterinary envenomations and fatalities [7]. The Israeli Ministry of Health is producing *V.x.p.* antivenom in horse based on a protocol developed by Moroz *et al*. The antigen consisting of 40% venom, 30% neurotoxic fraction and 30% hemorrhagic fraction together with adjuvant generating an efficient antivenom therapy [6,60,61,62]. Dose regiment of 50 mL of *V.x.p.* antivenom was reported to be efficacious in the treatment of systemic and progressive local manifestation caused by this snake envenomation [6].

Common side effects associated with its use and mild neutralization of all local or systemic effects of the venom are calling for modern studies to evaluate the precise proteomic composition and pathophysiological activity as well as development of more effective therapeutic strategies to deal with snake venom envenomation [63]. The Global Snakebite Initiative (GSI) is the development of multinational collaborative project to develop new regional polyvalent antivenoms in Asia and Africa using phylogenetic, proteomic and antivenomic tools to optimize immunogen selection in order to ensure broad multi snake coverage [64]. Therefore, this new approach calls for re-evaluation of proteomics and venomics of *V.x.p*. We hope that in the near future the second generation of poyvalent anti-*V.x.p.* venom under development by Kamada Ltd., Kiryat Weizmann, Rehovot, Israel will be found more efficacious and safe, and compatible with GSI polyvalent antivenom strategy.

## 6. Conclusions

There is a need for the development of efficient *V.x.p* polyvalent antivenom to treat envenomated patients in Israel. The development of this antivenom requires a modern understanding of the proteomics and pharmacological functions of the individual venom components. Re-evaluation of *V.x.p.* venom composition and function has been pursued in our laboratories over the last decade. Angiogenic factors, vascular endothelial like growth factors and nerve growth factors have been isolated and characterized. Viperistatin, a α1β1selective KTS disintegrin and VP12, a α2β1 selective C-type lectin were discovered. These snake venom proteins, proposed to represent novel hemorrhagins are promising tools for future research and development of novel drugs selective for integrins. In addition, the fact that new targets of antivenom therapy appear by more recent proteomic description of *V.x.p.* venom (such as the discovery of novel growth factors with hemorrhagic activities, of disintegrin and CLRPs), calls for re-evaluation of *V.x.p.* antivenom potential to neutralize these proteins. Full neutralization of all toxic components of *V.x.p.* venom is obligatory in order to achieve full protection of the patients.

## Conflict of Interest

The authors declare no conflict of interest.
